# Mobility of Rare Earth Elements in Coastal Aquifer Materials under Fresh and Brackish Water Conditions

**DOI:** 10.1021/acsenvironau.4c00001

**Published:** 2024-03-13

**Authors:** Nitai Amiel, Ishai Dror, Brian Berkowitz

**Affiliations:** Department of Earth and Planetary Sciences, Weizmann Institute of Science, Rehovot 7610001, Israel

**Keywords:** column experiments, humic acid, carbonates, batch experiments, complexation, rare earth elements

## Abstract

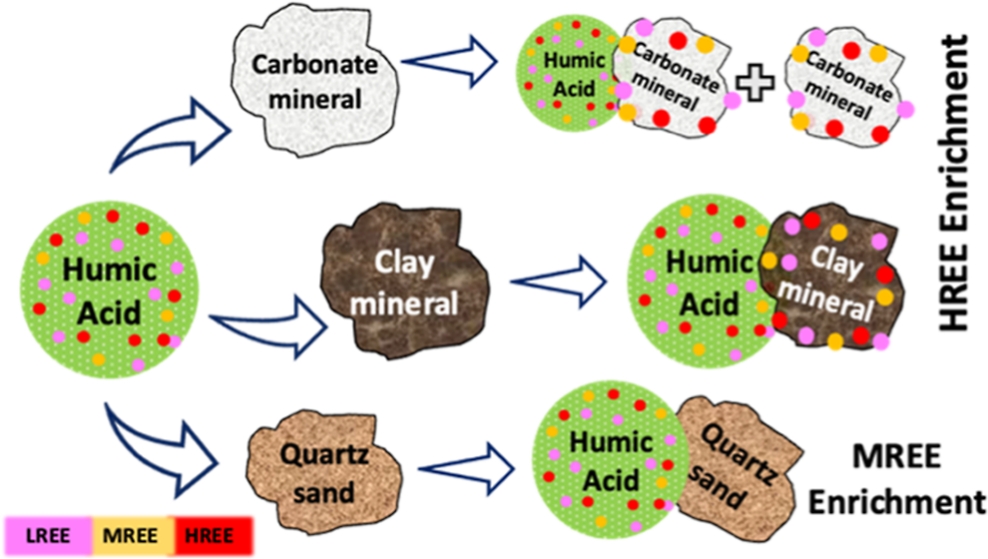

The indispensable role of rare earth elements (REEs) in manufacturing high-tech products and developing various technologies has resulted in a surge in REE extraction and processing. The latter, in turn, intensifies the release of anthropogenic REEs into the environment, particularly in the groundwater system. REE contamination in coastal aquifer systems, which serve as drinking and domestic water sources for large populations, demands a thorough understanding of the mechanisms that govern REE transport and retention in these environments. In this study, we conducted batch and column experiments using five representative coastal aquifer materials and an acid-wash sand sample as a benchmark. These experiments were conducted by adding humic acid (HA) to the REE solution under fresh and brackish water conditions using NaCl, representing different groundwater compositions in coastal aquifers. The REEs were shown to be most mobile in the acid-wash sand and natural sand samples, followed by two types of low-carbonate calcareous sandstone and one type of high-calcareous sandstone and the least mobile in red loamy sand. The mobility of REEs, found in solution primarily as REE–HA complexes, was controlled mainly by the retention of HA, which increases with increasing ionic strength and surface area of the aquifer material. Furthermore, it was found that the presence of carbonate and clay minerals reduces the REE mobility due to enhanced surface interactions. The higher recoveries of middle-REE (MREE) in the column experiment effluents observed for the acid-wash sand and natural sand samples were due to the higher stabilization of MREE–HA complexes compared to light-REE (LREE) and heavy-REE (HREE) HA complexes. Higher HREE recoveries were observed for the calcareous sandstones due to the preferred complexation of HREE with carbonate ions and for the red loamy sand due to the preferred retention of LREE and MREE by clay, iron, and manganese minerals.

## 1 Introduction

The rare earth element (REE) group comprises the lanthanide series elements (La–Lu; *Z* = 57–71) and Sc and Y (*Z* = 21 and 39, respectively). The different REEs have similar chemical and physical properties due to their equal number of electronic layers and similar electronic configurations. The main physical property that changes through the REE series is the cation radius, which decreases as the atomic number increases. The REE atomic structure gives them magnetic and spectroscopic properties, making them useful in many applications, particularly as crucial components in high-technology products.^1,2^ According to the International Energy Agency (IEA) report,^3^ the transition to clean energy by 2040 will increase the demand for REEs by 3.4 to 7.3 times compared to the demand in 2020 (according to the Stated Policies and the Sustainable Development scenarios, respectively). The demand for REEs in that context is primarily for electric vehicle motors and wind turbines, where REEs are used in manufacturing batteries and permanent magnets.^3^

The increasing mining and use of REEs in new and emerging technologies enhance the potential of REE release into the aquatic system. The primary sources of anthropogenic REE contamination in aquatic systems, mainly rivers and estuaries located next to large cities or industrial zones, are discharges from mining and mineral processing, disposal of industrial products, and effluents and wastewater from industrial processes that use REEs.^4^ Elevated concentrations of REEs in aquatic systems were reported in numerous countries (e.g., Japan,^5,6^ Poland,^7,8^ Brazil,^9^ South Korea,^10^ Germany,^11−14^ The Netherlands,^15^ Spain,^16^ France,^17^ USA,^18−20^ Israel,^21^ Australia,^22^ Switzerland,^23^ China,^24^ and the UK^25^).

The mobility of REE in aquatic systems is strongly influenced by their solution speciation, governed by the solution physicochemical properties: ionic strength, pH, redox potential, temperature, pressure, and the concentrations and type of organic and inorganic ligands.^26^ The processes that control the speciation of REE and the mobility of these species include water–solid sorption/desorption, coprecipitation with colloids, and complexation with organic and/or inorganic ligands.^2,27^ REEs are usually found in 3+ oxidation states in aquatic systems, although Europium (Eu) and Cerium (Ce) undergo changes in the oxidation state in specific environments. REE can form complexes with inorganic anions, such as carbonate, fluorine, phosphate, hydroxide, sulfate, and chloride, and with organic matter such as humic substances, for example, refs (1, 28–31). Generally, in circumneutral waters, REE complexation is dominated by humic substances (humic and fulvic acids). In contrast, in alkaline water with high carbonate concentration, REE also forms complexes with carbonate and bicarbonate ions.^32−34^

While the mechanisms that control anthropogenic REE mobility in rivers and estuaries were widely studied, for example, refs (35–37) relatively little is known about REE mobility in groundwater systems. Understanding the geochemical behavior of potential contaminants, such as anthropogenic REEs in aquifers, is critical because groundwater is a primary source of drinking water. So far, most studies on REE mobility in groundwater systems involved either (i) laboratory investigation on a single aquifer material (e.g., sand,^38−42^ soil,^43,44^ different clays,^45,46^ granite,^47^ and carbonate rock^48,49^), or (ii) analysis of complex field data, for example, refs (50–53). As a result, little is known about the mechanisms that control the mobility and retention of REEs while interacting with an aquifer system composed of complex combinations of natural materials, specifically, coastal aquifer materials.

A report by the UN states that ∼2.4 billion people, representing ∼40% of the world’s population, live within 100 km of the coast.^54^ Moreover, groundwater is estimated to supply 30–40% of global freshwater, with the rate of groundwater use growing rapidly for both drinking water and agriculture. Thus, understanding the potential contamination of coastal aquifers is critical. The chemical composition of coastal aquifer groundwater varies due to anthropogenic and natural processes (e.g., salinization, groundwater contamination from industries and agriculture, and water recharge to aquifers). These changes in the groundwater chemical composition affect the mobility of different solutes. For example, the changes in salinity and the concentration of organic matter affect REE speciation and retention mechanisms.^41,42^ In addition, the composition of the coastal aquifer might change spatially, which results in different retention mechanisms along paths of water flow.^53^

The main objective of this study is to explore the mechanisms that control the mobility and retention of REEs in different coastal aquifer materials at variable salinities. These goals are motivated by the fact that the production and use of REEs have grown significantly over the last decades, thus raising the potential for increased environmental contamination. This is particularly of high importance in the case of coastal aquifers, which represent major and often vulnerable water resource. In this context, we conducted a set of batch adsorption and transport column experiments in five representative aquifer materials composing the coastal aquifer of Israel under fresh and brackish water salinities. All retention experiments were conducted with the addition of humic acid to the REE solution. Humic acid is an important organic component of groundwater, as its presence enhances REE mobility while interacting with an aquifer material.^42^

## 2 Materials and Methods

### 2.1 Rock Sampling and Sample Handling

The coastal aquifer of Israel is composed of Pleistocene permeable calcareous sandstone (“Kurkar” group), interbedded with impermeable marine and continental silty clay lenses. The aquifer is typically porous, water-saturated, and aerobic. The “Kurkar” group extends from the surface to a depth of about 300 m.^55^ Five representative samples were sampled from outcrops of the “Kurkar” group. Sampling locations are detailed in Table S1 in the Supporting Information. The samples collected contained (1) natural sand, (2) two calcareous sandstone samples with low carbonate content, (3) calcareous sandstone with high carbonate content, and (4) red loamy sand. All samples were sieved through 0.5 mm mesh prior to the experiments.

An acid-washed quartz sand sample was used as a benchmark in all experiments. Quartz sand (mesh size 30/40), purchased from UNIMIN, USA, was washed with 5% nitric acid followed by a double-deionized water wash.

### 2.2 Reagents

A standard solution containing 10 mg L^–1^ La, Ce, Pr, Nd, Sm, Eu, Gd, Tb, Dy, Ho, Er, Tm, Yb, and Lu (IV-STOCK-26) was purchased from Inorganic Ventures. Sodium bromide (NaBr ≥ 99.5%), nitric acid (HNO_3_ 70%), and humic acid (HA) sodium salt were purchased from Sigma-Aldrich. REE solutions were prepared by diluting the REE standard in a 2 L flask and adding Br^–^ from a stock solution. Then, the solution pH was adjusted to pH 6, where >95% of all REEs in solution are present as REE^3+^, while the rest are present as REE–OH^2+^, using 0.1 M HCl and 0.1 M NaOH. Then, HA salt was added to a final concentration of 10 mg L^–1^, followed by a final pH adjustment to pH 8. REE speciation calculations were conducted using Visual MINTEQ version 3.1. The solutions were prepared 48 h prior to the onset of the different experiments to ensure complete complexation of REE with HA.^30^ The REE standard and the Br^–^ stock solution was diluted ×100 to reach concentrations of 100 μg L^–1^ for each REE and 1 mg L^–1^ Br^–^. All solutions were prepared using double deionized water (18.2 > MΩ cm).

### 2.3 Coastal Aquifer Material Characterization

The sand fraction (>0.063 mm) was determined using wet-sieve analysis, while the silt (0.002–0.06 mm) and clay (<0.002 mm) fractions were determined using the hydrometer method.^56^

Soil pH was measured at a soil-to-water ratio of 1:2 according to the protocol of Carter and Gregorich.^57^ Cation-exchange capacity was measured using the BaCl_2_ method.^58^

X-ray diffraction measurements were performed on Ultima III, Rigaku equipped with a sealed Cu tube operating at 40 kV/40 mA and a monochromator installed before the scintillator detector. Sollers of 2.5° were installed before and after the sample. Measurements were performed in Bragg–Brentano configuration within a range of 2–80° at a rate of 0.5°/min with a step of 0.02°. The measurements were performed on both the bulk sample and on a small-grain size fraction (<0.1 mm).

Total carbon and total organic carbon were measured using an elemental analyzer (FLASH 2000; Thermo Scientific). Total organic carbon was measured after removing calcareous carbonates from soil samples with HCl.^59^ The total inorganic carbon fraction was calculated by subtracting the total organic carbon fraction from the total carbon fraction.

### 2.4 Speciation Calculation

REE speciation in solutions containing 10 mg L^–1^ HA, at different IS (2.5 × 10^–3^ and 2.5 × 10^–2^ M), and pH 8 was calculated using the Stockholm Humic Model, integrated into Visual MINTEQ (Version 3.1^60^). Further discussion of the solution composition is given in Section 2.5. REE mobility was tested at pH 8 with HA concentrations of 10 mg L^–1^ due to the increased mobility of REEs in an HA-containing solution at alkaline pH and HA concentrations >5 mg L^–1^.^42^ The model applies a discrete-ligand approach that assumes that HA has eight proton-binding sites with distinct acid–base characteristics. Further details about the model can be found in Gustafsson^61^ and Gustafsson et al.^62^ Two parameters are needed to calculate REE speciation in the applied model: the intrinsic equilibrium constant for bidentate complexation (log *K*_Mb_) and the distribution term that modifies the strength of complexation sites (Δ*L*_K2_). As Gustafsson^61^ demonstrated, for trivalent cations (e.g., REEs), organic complexation is better fitted if only the bidentate binding sites are involved, excluding the monodentate complexation constant (log *K*_Mm_). The acid–base parameters for HA, the log *K*_Mb_, and the Δ*L*_K2_ values are detailed in Tables S2 and S3, at the Supporting Information. The acid–base parameters were set as generic model values from the “typicalha.mpf” database.^61^ The log *K*_Mb_ and the Δ*L*_K2_ were set according to Pourret et al.^63^ and Marsac et al.,^64^ respectively. The active-DOM/DOC ratio was set to 1.65 as the model default value. The speciation calculation showed complete (>99%) complexation of REEs with HA for the two ionic strengths tested.

### 2.5 Retention Experiments

Batch adsorption and column transport experiments were performed at pH 8 under two ionic strengths (fresh water: IS = 2.5 × 10^–3^, brackish water: IS = 2.5 × 10^–2^). It is noted that the pH was stable throughout the experiment within 0.1 pH units. The experimental solutions contained 100 μg L^–1^ of each REE, and Br^–^ tracer (1 mg L^–1^), 10 mg L^–1^ HA, and different NaCl concentrations according to the desired ionic strength. Although natural water contains additional ions, only Na and Cl were added to simplify the system and to examine solely the effect of salinity on the REE retention. It is further noted that 10 mg L^–1^ HA is a “realistic” organic matter concentration in domestic contaminants. In addition, previous studies^42^ showed that lower HA concentrations (5 mg L^–1^) do not allow the mobility of REEs and that 100 μg L^–1^ represents a “realistic” contaminant concentration that in extreme cases can become even much higher, for example, ref (65).

#### 2.5.1 Batch Experiments

Adsorption batch experiments were performed to quantify the retention of HA and REE on the five porous coastal aquifer materials at equilibrium. Two sets of experiments were conducted: the first with only HA in solution and the second contained both REE and HA. For each experiment, 0.5 L of the solution was mixed for 48 h and then placed in a flask containing 10 g of coastal aquifer material. Preliminary batch experiments showed that the optimal solid/solution ratio is 20 g/1 L. This ratio allows the retention kinetics to occur within days rather than hours or weeks. Each experiment was conducted in duplicate. The solutions were allowed to mix with soil on a rotating table and sampled hourly/daily for 8 days (as no changes in REE concentrations in the solution were detected after the eighth day). Control samples were taken before the solution was mixed with the soils to determine the initial concentrations of the REEs. Solution sampling was conducted several seconds after the experiment bottles were manually shaken, followed by sample filtration through a 0.22 μm filter. The sampled solutions were analyzed for REE concentrations using inductively coupled plasma mass spectrometry (ICP-MS) and for HA using UV absorption spectroscopy at λ = 218 nm (UV-1600; Shimadzu Corp.). Control samples of the REE solution without HA were analyzed for each aquifer material to ensure no lithological effect on HA measurements.

#### 2.5.2 Column Experiment

A set of vertical column experiments was conducted to study the mobility and retention of REEs under aerobic saturated flow. Two polycarbonate columns, 10 cm in length and width 1.5 cm in diameter, were packed for each coastal aquifer material. The flow in the columns was from bottom to top, via a multichannel peristaltic pump, in a fixed flow rate of 0.4 ± 0.01 mL min^–1^. The columns underwent saturation and pH adjustment phases by flowing pH-adjusted double deionized water in the desired ionic strength through the columns for 48 h prior to the experiment in a flow rate of 0.1 mL min^–1^. As the stabilization step ended, the double-deionized water was replaced with the REE–HA solution, and effluent collection at the column outlet began. After running the experimental solution for 44 h, the solution was switched back to double deionized water for 26 additional hours of column wash. Each experiment was carried out in duplicates. It is noted that because the work was done in closed columns under fully water-saturated conditions, there was no gas phase or direct contact with air; the experiments began after a long (48 h) equilibration period. In terms of redox conditions, the experiments can be considered very close to natural aquifer conditions.^66^ The collected samples were analyzed for the different REEs and Br concentrations by using ICP-MS. The Br tracer concentrations delineate the phase in which the REE-HA solution was injected. The water content (θ) of the column, packed with different coastal aquifer materials, was 0.135 ± 0.006, 0.195 ± 0.008, 0.142 ± 0.005, 0.148 ± 0.03, 0.152 ± 0.001, and 0.169 ± 0.002 for the acid-wash sand, natural sand, low-carbonate calcareous sandstone 1, low-carbonate calcareous sandstone 2, high-carbonate calcareous sandstone, and red loamy sand, respectively. Column experiments without HA addition to the REE solution were not conducted as REEs were previously shown to be immobile while flowing through quartz sand column.^42^

### 2.6 ICP-MS

All samples were analyzed via ICP-MS (Agilent 7700s) for the different REEs and Br^–^ concentrations. Drift corrections were carried out using indium as an internal standard and by repeatedly analyzing a calibration solution of 50 μg L^–1^ concentration as a drift monitor throughout the analysis. The recoveries of the internal standard and calibration solution were 100 ± 3% throughout the measurements. Memory effects were avoided by additional manual cleaning using 5% HNO_3_. To eliminate mass interferences for the different REEs, two isotopes were measured for every element when applicable. The following masses were measured: ^139^La, ^140^Ce, ^142^Ce, ^141^Pr, ^146^Nd, ^150^Nd, ^147^Sm, ^149^Sm, ^151^Eu, ^153^Eu, ^155^Gd, and ^157^Gd, ^159^Tb, ^163^Dy, ^164^Dy, ^165^Ho, ^166^Er, ^170^Er, ^169^Tm, ^172^Yb, ^174^Yb, ^175^Lu, and ^176^Lu.

### 2.7 Aqua Regia Digestion

The concentrations of REEs, iron, and manganese were measured by adding 10 mL of aqua regia to 0.5 g of powdered sample. After being kept at room temperature for 24 h, the samples were diluted with double deionized water to 50 mL and subsequently filtered using a 0.22 μm pore size filter. The procedure was conducted in triplicate for quality assurance. The digestion results and standard deviations are shown in Figure S1.

Then, the samples were further diluted 1:1 with double-deionized water. The measurements were done in duplicate for each sample.

## 3 Results and Discussion

Table 1 and Figures S2–S7, Supporting Information, demonstrate that the selected coastal aquifer materials display significant variations in their chemical and physical properties as well as mineralogical composition. Consequently, the mobility and retention of REEs in porous media are affected.

**Table 1:** Coastal Aquifer Material Properties^a^

aquifer material	pH	grain size (%)			TIC (%)	CEC cmol·kg^–^^1^
		sand	silt	clay		
AWS	7.90	100	0	0	<0.01	0.13 ± 0.05
NS	8.45	97	0	3	<0.01	0.42 ± 0.11
LCCS 1	8.85	95	2	3	1.62 ± 0.15	1.35 ± 0.08
LCCS 2	8.97	96	0	4	1.08 ± 0.38	1.29 ± 0.18
HCCS	9.22	84	12	4	5.89 ± 1.99	3.90 ± 0.38
RLS	8.88	81	6	13	0.03 ± 0.02	1.44 ± 0.33

a TIC—total inorganic carbon, CEC—cation exchange capacity, AWS—acid-wash sand, NS—natural sand, LCCS—low-carbonate calcareous sandstone; HCCS—high-carbonate calcareous sandstone; and RLS—red loamy sand.

The mobility and retention of REEs in the different coastal aquifer materials are discussed based on average REE recoveries in the different experiments (Table 3). The average REE recoveries were calculated by first calculating the recovery of each REE in a specific column experiment, and then calculating the average recoveries of all REEs present in the column experiment. REE recoveries in the batch adsorption experiment are presented as the relative concentrations of REEs in the aqueous phase after 8 days. Total REE recoveries for the column transport experiments were determined by calculating the fraction of each REE in the effluents by integrating the REE breakthrough curves (BTCs) from the total mass that entered the column.

Retention is defined as the mass of REEs not present in the solution for the batch experiments or the mass of REEs not eluted from the column in the flow experiments. Table 2 summarizes the retention of the total REEs expressed as the relative retained amount (in %) in the column compared to the sorption capacity at equilibrium observed for the corresponding batch system after 8 days. Comparisons of REE retention on all of the porous matrices under fresh and brackish water conditions for the column experiments, following the injection of 20, 50, 100, and 150 PV and for the batch system after 8 days, are reported in the Supporting Information as a set of 12 tables (Tables S5–S16). Details on how these values were calculated are also explained in the Supporting Information.

**Table 2:** Relative (%) Retained Total REEs in the Column Experiments Compared with the Equilibrium (Batch) Retention Capacity^a^

	20 PV	50 PV	100 PV	150 PV
AWS FW	5.1 ± 0.5	10.4 ± 0.9	18.9 ± 1.7	22.2 ± 2.0
AWS BW	3.5 ± 0.4	7.7 ± 0.9	14.9 ± 1.7	21.0 ± 2.3
NS FW	2.3 ± 0.2	5.1 ± 0.5	9.4 ± 0.9	13.5 ± 1.3
NS BW	2.3 ± 0.2	5.5 ± 0.6	10.3 ± 1.1	15.3 ± 1.6
LCCS 1 FW	2.4 ± 0.4	5.6 ± 0.9	10.8 ± 1.7	15.7 ± 2.5
LCCS1 BW	2.2 ± 0.4	5.4 ± 0.9	10.5 ± 1.8	14.9 ± 2.6
LCCS2 FW	2.5 ± 0.4	5.7 ± 1.0	11.1 ± 1.9	16.1 ± 2.8
LCCS2 BW	2.3 ± 0.4	5.7 ± 1.0	11.3 ± 2.0	16.8 ± 2.9
HCCS FW	1.8 ± 0.4	4.6 ± 1.1	8.2 ± 1.9	13.3 ± 3.1
HCCS BW	1.8 ± 0.5	4.5 ± 0.9	9.0 ± 1.7	13.0 ± 2.5
RLS FW	1.7 ± 0.4	4.2 ± 0.8	8.1 ± 1.4	12.1 ± 2.2
RLS BW	1.7 ± 0.2	4.3 ± 0.3	8.4 ± 0.5	12.7 ± 0.8

a AWS—acid-wash sand; NS—natural sand; LCCS—low-carbonate calcareous sandstone; HCCS—high-carbonate calcareous sandstone; RLS—red loamy sand FW—fresh water; and BW—brackish water.

**Figure 1:**
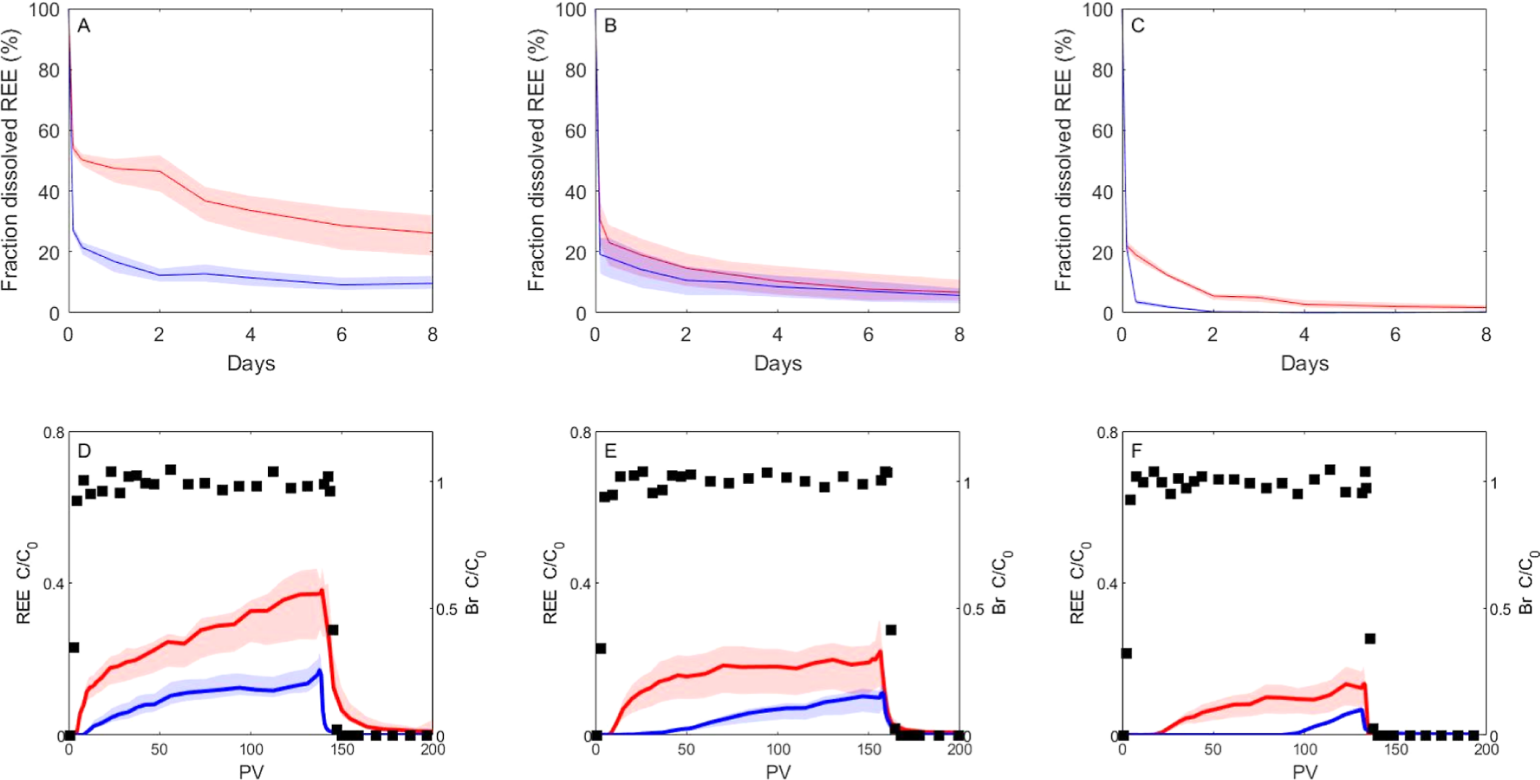
Adsorption curves (A–C) and breakthrough curve measurements (D–F) of REEs (average concentration) in representative Coastal Aquifer materials and salinities. (A,D) Natural sand, (B,E) low-carbonate calcareous sandstone #2, and (D,F) red loamy sand. Red line: REE average in fresh water conditions (IS = 2.5 × 10^–3^ M). Red background: REE distribution in fresh water conditions. Blue line: REE average in brackish water conditions (IS = 2.5 × 10^–2^ M). Blue background: REE distribution in brackish water conditions. Black squares: Br tracer.

Figure 1 shows REE adsorption isotherms and BTCs of three representative coastal aquifer materials, while the entire set of results is presented in Figures S8 and S9, Supporting Information. Generally, REEs were more mobile in acid-wash sand, followed by natural sand, low-carbonate calcareous sandstones, high-carbonate calcareous sandstones, and red loamy sand. In addition, REE mobility for a specific coastal aquifer material was higher for fresh water than for brackish water conditions (IS 2.5 × 10^–3^ and 2.5 × 10^–2^ M, respectively). This trend was similar for batch (shown as a higher concentration of REEs in the aqueous phase) and column experiments (Table 3). Furthermore, variations in REE fractionation pattern, the difference in their recoveries along the REE series, were also observed between the different coastal aquifer materials.

**Table 3:** Average Recoveries of REEs in the Different Retention Experiments under Fresh Water and Brackish Water Conditions for Different Coastal Aquifer Materials^a^

aquifer material	column experiments (%)		batch experiment (%)	
	fresh water	brackish water	fresh water	brackish water
AWS	48.4 ± 4.3	28.1 ± 3.1	67.4 ± 6.8	52 ± 4.3
NS	28.4 ± 2.8	13.9 ± 1.4	29.6 ± 2.2	25.2 ± 1.8
LCCS 1	16.6 ± 2.7	4.6 ± 0.8	28.9 ± 5.3	19.4 ± 1.4
LCCS 2	14.9 ± 2.9	8.4 ± 1.0	31.2 ± 6.4	24.4 ± 2.5
HCCS	3.9 ± 0.9	3.7 ± 0.8	6.4 ± 2.1	5.4 ± 1.7
RLS	7.2 ± 1.3	1.4 ± 0.1	1.7 ± 0.4	0.27 ± 0.1

a AWS—acid-wash sand; NS—natural sand; LCCS—low-carbonate calcareous sandstone; HCCS—high-carbonate calcareous sandstone; and RLS—red loamy sand.

In conjunction with the findings in Table 2 (and Tables S5–S16), the BTC in Figure 1 illustrates that the REEs exhibit transport and elution from the column even though the sorption capacity has not been exhausted. This observed mobility underscores the distinction between batch experiments, which capture the equilibrium sorption capacity of the matrix, and the dynamic behavior of the flow system. The latter is more pertinent to real-world conditions, where equilibrium is seldom achieved. Notably, in our specific case, the elution of REEs from the column commences before 20 PV, corresponding to less than 5% sorption capacity. It is further noted that the differences in the elution pattern between fresh and brackish water are not correlated to the similar sorption capacity observed in both cases, suggesting that the mobility is much more sensitive to the solubilities of the REEs than the sorption capacity of the solid matrix.

REE retention mechanisms (Section 3.1), the effect of the aquifer material properties, and the salinity change on REE mobility are discussed (Sections 3.2 and 3.3), based on the comparison between the average recovery of the different REEs for every aquifer material and salinity examined. The effect of aquifer material properties on the REE fractionation pattern is discussed separately based on the recoveries of the different REEs (Sections 3.4).

### 3.1 REE Speciation and Retention Mechanism

The retention of the different REEs while interacting with an aquifer material strongly depends on REE speciation and the aquifer material properties. Speciation calculation shows that all REEs (>99%) are complexed with HA in fresh and brackish water conditions (for ionic strength 2.5 × 10^–3^ and 2.5 × 10^–2^ M, respectively).

In the ternary system containing REE, HA, and minerals, the retention of REEs could result from the coretention of REE and HA as REE-HA complex on the mineral surface or from the retention of REE^3+^ solely, following REE dissociation from the HA complex.^41,42,67^ To better understand the retention mechanism of the REE–HA complex on the different coastal aquifer materials, at pH 8, under fresh and brackish water conditions, the ternary system was examined as two binary systems. The first includes the retention of HA in an REE-free solution, while the second includes the retention of REE in an HA-free solution (REE^3+^).

Humic acid retention on the different coastal aquifer materials was examined in a batch adsorption experiment in an REE-free solution (HA-mineral binary system). HA retention increased as a function of ionic strength for all coastal aquifer materials under fresh and brackish water conditions (Figure S10). The HA retention in a REE-free solution was the highest on red loamy sand and high-carbonate calcareous sandstone, followed by low-carbonate calcareous sandstones, natural sand, and acid-wash sand (Figure S10). The observed HA retention order is highly correlated with the retention of REEs in an HA-containing solution (Table 2). The effect of ionic strength on HA retention is discussed in Section 3.3.

Contrary to the above observations, previous studies have shown that the retention of REE^3+^ in a solution without HA (in REE-minerals binary system) decreases as the ionic strength increases.^40,41^ The reason for the decreased retention of REE with increasing ionic strength is attributed mainly to the reduction in exchange sites for REE surface exchange reactions, brought about by the presence of the electrolyte cation.^40^

The matching between the REE retention order in a solution that contains HA and the retention of HA in an REE-free solution implies the coretention of REE and HA in the form of REE–HA complexes. Moreover, this similarity demonstrates that the retention of HA largely dominates the retention of REE and REE–HA complexes, although a minor amount of dissociated REE^3+^ may also be retained.

### 3.2 Effect of Coastal Aquifer Material Properties on REE Transport and Retention

The retention and mobility of REEs in coastal aquifer porous materials varied greatly due to their varying characteristics, as shown in Tables 1–3. Clay and carbonate minerals, the two mineral factors that impact the retention of REEs and HA in porous media,^45,68^ exhibit significant differences among the various coastal aquifer materials.

To assess the influence of carbonate and clay minerals on REE-HA retention, the coastal aquifer materials were separated into two overlapping groups. The first group of coastal aquifer materials, comprising natural sand, low-carbonate calcareous sandstones, and high-carbonate calcareous sandstone, have varying levels of inorganic carbonate content and a similar fraction of clay-size particles (3–4%; Table 1). The second group of coastal aquifer materials, comprising the acid-washed sand, natural sand, and red loamy sand samples, have varying amounts of clay-sized particles and similar low content of inorganic carbonates (<0.01%; Table 1).

The retention of REE in the first group of aquifer materials, which contains different fractions of inorganic carbonate, was the highest for the high-carbonate calcareous sandstone, followed by the low-carbonate calcareous sandstones and the natural sand sample (Table 2). The natural sand sample contains a minute amount of inorganic carbonate and exhibits the lowest retention (highest recoveries). Organic carbon concentrations in all aquifer materials tested in this study were lower than 0.01%.

The two low-carbonate calcareous sandstones contain 1.35 ± 0.38% of inorganic carbonate and exhibit higher retention than the natural sand sample but lower than the high-carbonate calcareous sandstone sample, which contains 3.9 ± 0.38% inorganic carbonate. The order of REE retention in those aquifer materials agrees with their inorganic carbonate content (Table 1).

The retention of REE in the second group of aquifer materials, containing different fractions of clay-size particles, was the highest for the red loamy sand, followed by natural sand and acid-wash sand (Table 2).

The acid-wash sand sample, which exhibits the lowest REE retention (highest recoveries), is composed entirely of quartz sand particles (Table 1 and Figure S2). The natural sand sample, which exhibits higher REE retention than the acid-wash sand, contains 3% clay-size particles (Table 1 and Figure S3). The red loamy sand sample, which exhibits the highest REE retention, contains 13% clay-size particles composed mainly of smectite (Table 1 and Figure S7). The clay-size fraction in those aquifer materials agrees with the trend of the REE retention in those materials.

As shown in Section 3.1, the retention of REE in an HA-containing solution is governed mainly by the retention of HA on the different coastal aquifer materials, resulting in the retention of REE–HA complexes. The adsorption of HA on the surface of different minerals was studied by Petrović et al.,^68^ which showed that at pH > 7, HA retention on the surface of clay and carbonate minerals (calcite) is higher than in sand. Thus, the higher retention of HA on carbonate and clay minerals results in an increased retention of REEs on coastal aquifer materials that comprise of significantly higher fractions of carbonate and clay-sized minerals, as REEs are coretained with HA.

The retention of HA and REE-HA complexes on different minerals is controlled mainly by the accessibility of active sites on the mineral surface, which is a function of the surface area of the mineral. As more HA is bound to the mineral surface, the accessibility to active sites on the mineral surface decreases due to steric blocking by the HA molecules.^68^ The trend of REE retention agrees with the increasing portion of clay-sized particles in the samples (Tables 1 and 2).

### 3.3 Ionic Strength Effect on REE Retention and Mobility

REE retention on the different aquifer materials, which was shown to be controlled mainly by HA retention (Section 3.1), was higher under brackish water conditions (higher ionic strength) than under fresh water conditions for all coastal aquifer materials (Figures 1, S8, and S9 and Table 2). Wan and Liu^67^ and Yoshida and Suzuki^41^ reported similar behavior, namely, higher retention of REE in an HA-containing solution on kaolin and sand, respectively, when the solution ionic strength increased. The higher retention of HA at higher ionic strength, at pH 8, could be due to (1) increased hydrophobicity of HA with higher ionic strength,^67^ (2) weaker electrostatic repulsion between the adsorbed HA and the negatively charged mineral surface related to the effect of the electrolyte cation,^69^ and (3) smaller size of HA molecules as ionic strength increases due to aggregation,^70^ which in turn enable more HA adsorption on the mineral surface.^71^ At higher ionic strengths, when the size of the HA molecules is smaller than in lower ionic strength, the HA molecules can easily penetrate the structured first few layers of adsorbed water near the mineral surface (the “electric double layer”), which results in higher HA retention.^69^

In the ternary system composed of HA, REE, and minerals, an increase in ionic strength would result in stronger competition from the countercation, leading to greater aggregation of HA. The aggregation of HA reduces the availability of ligands for complexation with REE and decreases the strength of the interactions between HA and REE.^33,72^ Consequently, at similar pH (pH 8) and higher ionic strength, the reduced complexation availability of the smaller HA molecules leads to a higher number of HA molecules that form complexes with REEs. Due to the smaller size of the HA molecules, the steric effect that limits HA retention on the particle surface decreases and more REE-bound HA molecules are retained on the mineral surface. The increased REE retention at high ionic strength implies that reducing the steric effect is more pronounced than the complexation availability of the HA molecules, as HA concentrations (10 μg L^–1^) are much higher than REE concentrations (total REE: 140 μg L^–1^). In addition, the decreased complexation availability of the HA molecules could lead to a decrease in the amount of HA-complexed REEs, and an increase in the amount of REE^3+^ ions. The increase in REE^3+^ in solution is expected to be minor, as HA was shown to govern REE retention at high ionic strength. This, in turn, will increase REE retention, as the retention of REE^3+^ is more robust than that of REE–HA molecules at pH 8.^40,42,45^

### 3.4 REE Fractionation Pattern

The REE fractionation pattern results from the gradual decrease in the REE cation radius with increasing atomic number.^73^ This change affects REE interaction with ligands and other organic and inorganic materials, leading to different speciation and consequently retention along the REE series.^26^ Furthermore, due to the varying mineral compositions of rocks and soils, there will be differences in the patterns of REE fractionation, arising from variations in the distribution of REE between the solid and the solution during interaction with the solid matrices.^74^

**Figure 2:**
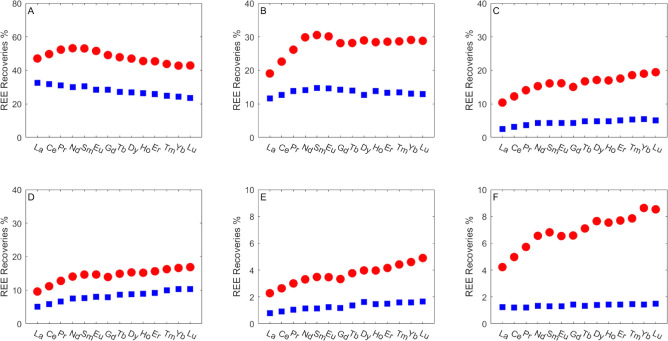
REE recovery patterns in column transport experiments for different coastal aquifer materials and salinities. (A) Acid-wash sand, (B) natural sand, (C) low-carbonate calcareous sandstone 1, (D) low-carbonate calcareous sandstone 2, (E) high-carbonate calcareous sandstone, and (F) red loamy sand. LREEs—La, Ce, Pr, and Nd, MREEs—Sm, Eu, and Gd, HREEs—Tb, Dy, Ho, Er, Tm, Yb, and Lu; Red dots: fresh water conditions (IS = 2.5 × 10^–3^ M). Blue dots: brackish water conditions (IS = 2.5 × 10^–2^ M).

In this study, the REE fractionation pattern was examined with respect to the recoveries of the different REEs in the column transport experiments. The BTC of each REE within a single experiment was integrated to yield the total recovery (Figure 2). The REE concentrations in the different aquifer materials, measured by aqua regia digestion, are in the range of 0.1–10 μg kg^–1^. Based on the initial low REE concentrations, the difference between REE fractionation patterns in the aquifer materials and in the eluted solution, and the relatively high pH (pH 8), which does not favor REE leaching from the rock, we consider the natural REE abundance in the rock to have an insignificant effect on the observed REE fractionation pattern.

Due to the effect of clay and carbonated minerals on REE retention (Section 3.2), the discussion dealing with REE fractionation pattern would focus on two groups of aquifer material: (1) the sand samples, which contain only a minute amount of inorganic carbonate (acid-wash sand, natural sand, and red loamy sand), and (2) the calcareous sandstones, which contain substantial inorganic carbonate content (Table 1). To characterize the REE fractionation pattern, three selected elements, La, Sm, and Yb, were used to represent the light-REE (LREE), middle-REE (MREE), and heavy-REE (HREE) forms, respectively. The complete set of REEs included the LREEs La, Ce, Pr, and Nd, the MREEs Sm, Eu, and Gd, and the HREEs Tb, Dy, Ho, Er, Tm, Yb, and Lu. The lower/higher recoveries of HREE relative to LREE or MREE (depletion/enrichment, respectively) would yield different Yb/La and Yb/Sm ratios, respectively. For example, higher recoveries of HREE, compared to MREE, yield Yb/Sm > 1.

For the first group of aquifer materials that contain only a minute amount of inorganic carbonate, higher MREE recoveries were observed for the acid-wash sand and natural sand, while higher HREE recoveries were observed for the red loamy sand (Figure 2A,B,F, respectively). The high recoveries of MREE compared to LREE and HREE observed for the acid-wash sand (Figure 2A) and natural sand (Figure 2B) are related to the significant complexation of MREE with HA.^30^ The MREE-HA complexes are more stable in solution than LREE–HA and HREE–HA complexes,^30^ which leads to a reduced retention of MREE in sand samples, as reported by Amiel et al.^42^ for acid-wash sand. Although both acid-wash sand and natural sand samples showed higher MREE recoveries (Yb/Sm < 1), natural sand has a higher HREE/MREE ratio (Yb/Sm = 0.95, 0.89 for fresh and brackish water, respectively) than acid-wash sand (Yb/Sm = 0.80, 0.79 for fresh and brackish water, respectively). The acid-wash sand and natural sand samples are composed of quartz sand (Figures S2 and S3), except for a small fraction (3%) of clay particles in the natural sand (Table 1).

REE fractionation pattern in the red loamy sand sample, which contains the most significant fraction of clay particles (13%), shows higher recoveries of HREE compared to MREE for both fresh and brackish water conditions (Yb/Sm = 1.26 and 1.09 for fresh and brackish water, respectively). From those observations, it can be concluded that for aquifer materials with a minute amount of carbonate, a higher fraction of clay-size particles would increase the recoveries of HREE over LREE and MREE, but generally decrease the average REE recoveries (Section 3.2). Clay minerals were previously shown to produce higher HREE recoveries pattern at HA concentrations >5 mg L^–1^ due to higher retention of LREE and MREE on clays than HREE.^67^

The preferential scavenging of LREE and MREE by Fe and Mn minerals is another mechanism that could result in increased HREE recoveries. Koeppenkastrop and De Carlo,^75^ found in their batch adsorption experiments reduced adsorption for HREEs onto MnO_2_ and FeOOH when examining the adsorption of REEs from solution by metal oxides. Davranche et al.^76^ observed increased LREE adsorption on MnO_2_ in an HA-containing solution due to the dissociation of REE from the REE-HA complex. Although REE retention was shown to be controlled mainly by HA retention, as REE and HA were coretained as REE–HA complexes (Section 3.1), minor REE dissociation could occur. REE dissociation from humic molecules was previously reported to occur when a highly competitive ligand is present. In this case, REEs from the less stabilized REE–HA complexes could dissociate and redistribute. This redistribution includes the reabsorption onto solid minerals (such as Fe and Mn minerals) or the formation of a new complex with a competitive ligand.^76^ The fraction of Fe and Mn, well-known REE scavengers, in the different aquifer materials was measured using aqua regia digestion (Figure S11). Results show that Fe and Mn concentrations are the highest for the red loamy sand, followed by the natural sand and acid wash sand. Consequently, the increased Fe and Mn concentrations in the sample would promote higher HREE recoveries due to the preferential scavenging of LREE and MREE.

The REE pattern for the calcareous sandstone samples, which compose the second group of aquifer materials, shows higher recoveries of HREE compared to LREE and MREE. The HREE/MREE ratio was higher for high-carbonate calcareous sandstone (Yb/Sm = 1.32 and 1.40 for fresh and brackish water, respectively) than for low-carbonate calcareous sandstone 1 (Yb/Sm = 1.18 and 1.28 for fresh and brackish water, respectively) and low-carbonate calcareous sandstone 2 (Yb/Sm = 1.18 and 1.28 for fresh and brackish water, respectively).

For this group of aquifer materials, the recoveries of HREE increase with increasing inorganic carbonate concentrations in the samples, which could enter the solution as carbonate ions. Tang and Johannesson^40^ reported a similar REE adsorption pattern in the presence of elevated *P*_CO_2__ (≥10^–2.3^ atm), as HREE adsorption on sand was lower compared to LREE and MREE. Carbonate ions have a higher affinity toward HREE than LREE and MREE in natural alkaline water, resulting in HREE partitioning between HA and carbonate complexes, while LREE and MREE are complexed with HA.^34^ Consequently, we suggest that the carbonate minerals serve as a source of carbonate ions, which in turn are complexed with HREE and increase their mobility relative to those of MREE and LREE. Here, we show that higher concentrations of carbonate minerals in the aquifer material result in higher HREE recoveries. These observations are applicable only at low, environmentally representative REE concentrations, as conducted in this study. An increase in REE concentration by several orders of magnitude could result in precipitation on REE-carbonate minerals.

### 3.5 Coastal Aquifer Perspective

The geochemical implication of our results is that the retention of REE contamination, containing high organic matter concentrations, in porous natural coastal aquifers is likely to be higher at the fresh water low-salinity zone and lower at the mixing, high-salinity zone. REE mobility will decrease when flowing through an aquifer with a high clay and inorganic carbonate content.

## 4 Conclusions

Considering the elevated potential for release of contamination plumes that contain high concentrations of REEs, we investigated the mechanisms that control the mobility and retention of REEs in different coastal aquifer materials: natural sands, low- and high-carbonate calcareous sandstones, and red loamy sand, with consideration of an acid-washed sand sample serving as a benchmark. REE recoveries, an indicator of REE mobility, were higher for acid-wash and natural sand samples, followed by calcareous sandstones and finally red loamy sand.

The REE–HA complexes were coretained on the aquifer materials, and the HA retention on the different aquifer materials governed the process rather than REE retention. HA retention on different aquifer materials is controlled by the steric effect. The number of accessible active sites on the mineral surface changes as a function of the surface area of the mineral and the size of the HA molecules. Coastal aquifer materials with a high fraction of clay-size particles exhibit a higher surface area, which decreases the steric effect and promotes the retention of HA and, thus, HA-complexed REEs. The ionic strength of the solution changed the size of the HA molecules. The higher ionic strength condensed the HA and decreased its molecular size. This, in turn, reduced the amount REE bound to an HA molecule but also reduced the steric effect, which allowed more REE-bound HA retention on a certain coastal aquifer material. The increasing REE retention at higher ionic strength suggests that lowering the steric effect is more dominant than lowering the complexation availability of the HA molecules.

REE fractionation patterns varied among the coastal aquifer materials. The higher recoveries of MREE in the sand samples are due to higher stabilization of the MREE–HA complexes, which increases the MREE mobility. The higher recoveries of HREE observed in the calcareous sandstones are due to the presence of carbonate minerals, which released carbonate ions into the solution. The higher recoveries of HREE observed in the red loamy sand may be attributed to enhanced LREE and MREE retention by clay minerals. Additionally, the high concentrations of Fe and Mn minerals could also play a role in the preferential scavenging of LREE and MREE over HREE, leading to higher recoveries of HREE in the red loamy sand.

## Supporting Information Available

Sampling locations of the different coastal aquifer materials, list of constants used for REE speciation using Stockholm Humic Model, list of logKMb and ΔLK2 values used for REEs speciation calculations using Stockholm Humic Model, retained REE mass per 1 g of coastal aquifer materials in fresh and brackish water, concentrations of REEs in the different coastal aquifer materials, XRD measurements of all six examined samples, batch adsorption experiment results, column transport experiment results, Humic acid retention on the different coastal aquifer materials in fresh and brackish water, and concentrations of Mn and Fe in the different coastal aquifer materials (PDF)

## Abbreviations

BTC: breakthrough curve
HA: humic acid
PV: pore volume
REE: rare earth elements
