# Supplementary material for: Mobility of Rare Earth Elements in Coastal Aquifer Materials under Fresh and Brackish Water Conditions
Source: ACS Environ Au. 2024 Mar 13;4(4):186–95. doi: 10.1021/acsenvironau.4c00001 (PMC11258752; doi:10.1021/acsenvironau.4c00001)
Supplement: Supplementary file 1 — vg4c00001_si_001.pdf [file vg4c00001_si_001.pdf]

Supporting Information

Mobility of Rare Earth Elements in coastal aquifer materials under fresh and brackish water conditions

Nitai Amiel*[1], Ishai Dror[1], and Brian Berkowitz[1]

[1]Department of Earth and Planetary Sciences, Weizmann Institute of Science, Rehovot 7610001, Israel

The Supporting Information contains figures and tables showing the sampling locations of the different coastal aquifer materials (Table S1), a list of constants used for REE speciation using Stockholm Humic Model (Table S2), a list of logK_{Mb} and ΔLK_2 values used for REEs speciation calculations using Stockholm Humic Model (Table S3),. Retained REE mass per 1 g of coastal aquifer materials in fresh and brackish water (Tables S5-S16). The Concentrations of REEs in the different coastal aquifer materials (Figure S1); XRD measurements of all six examined samples are presented in Figures S2-7, batch adsorption experiment results (Figure S8), and column transport experiment results (Figure S9), Humic acid retention on the different coastal aquifer materials in fresh and brackish water (figure S10), Concentrations of Mn and Fe in the different coastal aquifer materials (Figure S11).

Table S1. Coastal aquifer porous media sampling locations.

Sample	Latitude	Longitude
Acid-wash sand	NA	NA
Natural sand	31°51'37.20"N	34°48'08.90"E
Low-carbonate calcareous sandstone 1	31°55'55.47"N	34°42'57.38"E
Low-carbonate calcareous sandstone 2	31°55'26.60"N	34°46'34.60"E
High-carbonate calcareous sandstone	31°55'56.33"N	34°42'56.94"E
Red sandy soil	31°55'26.60"N	34°46'34.60"E

Table S2. List of constants used for REE speciation using Stockholm Humic Model.

Parameter Description Values		
n_A	Amount of type-A sites (mol g^{-1})	3.55E-03
n_B	Amount of type-B sites (mol g^{-1})	1.78E-03
log K_A	Intrinsic proton dissociation constant for type-A sites	-4.13
log K_B	Intrinsic proton dissociation constant for type-B sites	-8.99
ΔpK_A	Distribution term that modifies log K_A	3.03
ΔpK_B	Distribution term that modifies log K_B	3.03
log K_{Mb}	Intrinsic equilibrium constant for bidentate complexation of metal M	Table S3
ΔLK_2	Distribution term that modifies the strength of complexation sites	Table S3
r	Molecular radius	1.8 nm
C	Stern layer capacitance	2 F m^{-2}
Ns	Site density of HS functional groups	1.2 sites nm^{-2}
gf	Gel fraction parameters	0.78
K_C	Intrinsic equilibrium constant for accumulation of screening counterions	$10^{0.8}$

Table S3. logK_{Mb} and ΔLK_2 values for REEs speciation calculations using Stockholm Humic Model.

REE	logK_{Mb}[a]	ΔLK_2[b]
La	-5.66	1.1
Ce	-5.6	1.15
Pr	-5.13	1.2
Nd	-5.04	1.2
Sm	-4.65	1.3
Eu	-4.79	1.35
Gd	-5.04	1.35
Tb	-5.4	1.5
Dy	-5.5	1.6
Ho	-5.57	1.65
Er	-5.65	1.7
Tm	-5.71	1.9
Yb	-5.3	2.05
Lu	-5.5	2.1

[a] Pourret et al. (2007)

[b] Marsac et al. (2010)

Comparing REE retention between batch and column experiments

The retained REEs were calculated for column experiments (after 20, 50, 100 and 150 PV) and for batch experiments (after 8 days).

Method of calculation:

Batch experiments:

The concentrations of retained REEs (µg REE/ gr soil) were calculated by first measuring the REE concentrations in solution after 8 days and subtracting the REEs in the solution from the initial REE concentrations. The retained REE concentrations were multiplied by the solution volume to determine the retained REE mass. The retained REE mass was divided by the soil mass in the experimental bottles. An average (and standard deviation), "representative" REE concentration, based on all REE concentrations, was then determined.

Data:

- Initial REE concentrations: 70 µg/L for each REE
- Volume of solution: 500 mL
- Mass of soil in experimental bottles: 10 g

Column experiments:

The concentration of retained REEs (µg REE/ gr soil) in the column after injecting 20, 50, 100, and 150 PV was calculated by measuring the mass of soil in each column and calculating the PV of each column by subtracting the unsaturated weight of the column from its saturated weight. The volume of solution that flows through the column after 20, 50, 100, and 150 PV was calculated by multiplying the number of PV by the volume (mL) of one PV of the column. The mass of REEs that entered the column was calculated by multiplying the initial REE concentrations by the volume of solution that flowed through the column.

The recoveries of REEs (mass) after injecting a given number of PV were determined by integrating their BTCs. The retained REE mass was calculated by subtracting the recovered REEs from the total REEs that entered the column. The retained REE for a g of soil was determined by dividing the retained REE mass by the soil mass.

Data:

- Initial REE concentrations: 70 µg/L for each REE
- PV and soil mass for the different aquifer materials are presented in Table S4

Table S4. Measured soil mass and PV for aquifer materials used in the experiments.

**Aquifer material**	**mass of soil (g)**	**PV (mL)**
Acid-wash sand	75	8.2
Natural sand	79	7.4
Low-carbonate calcareous sandstone 1	79	6.8
Low-carbonate calcareous sandstone 2	80	6.9
High-carbonate calcareous sandstone	81	7.1
Red loamy sand	82	7.9

Table S5. Retained REE mass per 1 g of acid-wash sand for batch and column experiments with fresh water.

**Acid-wash sand Fresh water**	Retained REE (µg REE / g soil)				
	Column exp				Batch exp
	**20 PV**	**50 PV**	**100 PV**	**150 PV**	**8 Days**
La	1.11E-01	2.28E-01	4.13E-01	4.85E-01	2.19E+00
Ce	1.34E-01	2.74E-01	4.97E-01	5.84E-01	2.63E+00
Pr	1.23E-01	2.53E-01	4.59E-01	5.39E-01	2.43E+00
Nd	1.25E-01	2.57E-01	4.67E-01	5.48E-01	2.47E+00
Sm	1.25E-01	2.57E-01	4.66E-01	5.46E-01	2.47E+00
Eu	1.22E-01	2.50E-01	4.53E-01	5.31E-01	2.40E+00
Gd	1.16E-01	2.38E-01	4.31E-01	5.06E-01	2.28E+00
Tb	1.13E-01	2.31E-01	4.20E-01	4.92E-01	2.22E+00
Dy	1.11E-01	2.28E-01	4.12E-01	4.84E-01	2.18E+00
Ho	1.07E-01	2.20E-01	4.00E-01	4.69E-01	2.12E+00
Er	1.07E-01	2.20E-01	3.98E-01	4.67E-01	2.11E+00
Tm	1.03E-01	2.12E-01	3.84E-01	4.51E-01	2.03E+00
Yb	1.01E-01	2.07E-01	3.75E-01	4.40E-01	1.99E+00
Lu	1.01E-01	2.08E-01	3.76E-01	4.41E-01	1.99E+00
**Average**	1.14E-01	2.34E-01	4.25E-01	4.99E-01	2.25E+00
**STD**	1.02E-02	2.10E-02	3.80E-02	4.46E-02	2.01E-01

Table S6. Retained REE mass per 1 g of acid-wash sand for batch and column experiments with brackish water.

**Acid-wash sand Brackish water**	Retained REE (µg REE / g soil)				
	Column exp				Batch exp
	**20 PV**	**50 PV**	**100 PV**	**150 PV**	**8 Days**
La	1.34E-01	2.95E-01	5.75E-01	8.07E-01	3.85E+00
Ce	1.39E-01	3.07E-01	5.97E-01	8.38E-01	3.99E+00
Pr	1.27E-01	2.81E-01	5.46E-01	7.67E-01	3.66E+00
Nd	1.23E-01	2.72E-01	5.30E-01	7.44E-01	3.55E+00
Sm	1.25E-01	2.76E-01	5.37E-01	7.54E-01	3.59E+00
Eu	1.17E-01	2.57E-01	5.01E-01	7.03E-01	3.35E+00
Gd	1.17E-01	2.58E-01	5.02E-01	7.05E-01	3.36E+00
Tb	1.12E-01	2.46E-01	4.79E-01	6.73E-01	3.21E+00
Dy	1.11E-01	2.44E-01	4.75E-01	6.67E-01	3.18E+00
Ho	1.08E-01	2.39E-01	4.64E-01	6.52E-01	3.11E+00
Er	1.06E-01	2.35E-01	4.57E-01	6.42E-01	3.06E+00
Tm	1.02E-01	2.25E-01	4.37E-01	6.14E-01	2.93E+00
Yb	9.94E-02	2.19E-01	4.27E-01	5.99E-01	2.86E+00
Lu	9.62E-02	2.12E-01	4.13E-01	5.80E-01	2.77E+00
**Average**	1.15E-01	2.55E-01	4.96E-01	6.96E-01	3.32E+00
**STD**	1.29E-02	2.85E-02	5.55E-02	7.79E-02	3.72E-01

Table S7. Retained REE mass per 1 g of natural sand for batch and column experiments with fresh water.

**Natural sand Fresh water**	Retained REE (μg REE / g soil)				
	Column exp				Batch exp
	**20 PV**	**50 PV**	**100 PV**	**150 PV**	**8 Days**
La	7.59E-02	1.69E-01	3.14E-01	4.51E-01	3.34E+00
Ce	1.16E-01	2.58E-01	4.79E-01	6.88E-01	5.09E+00
Pr	1.04E-01	2.33E-01	4.32E-01	6.20E-01	4.58E+00
Nd	1.19E-01	2.66E-01	4.93E-01	7.07E-01	5.23E+00
Sm	1.22E-01	2.71E-01	5.04E-01	7.23E-01	5.35E+00
Eu	1.20E-01	2.68E-01	4.97E-01	7.14E-01	5.28E+00
Gd	1.12E-01	2.50E-01	4.64E-01	6.66E-01	4.93E+00
Tb	1.12E-01	2.50E-01	4.65E-01	6.67E-01	4.94E+00
Dy	1.15E-01	2.57E-01	4.78E-01	6.86E-01	5.07E+00
Ho	1.13E-01	2.53E-01	4.69E-01	6.73E-01	4.98E+00
Er	1.14E-01	2.54E-01	4.71E-01	6.76E-01	5.00E+00
Tm	1.14E-01	2.55E-01	4.73E-01	6.79E-01	5.02E+00
Yb	1.16E-01	2.59E-01	4.80E-01	6.89E-01	5.09E+00
Lu	1.15E-01	2.56E-01	4.75E-01	6.82E-01	5.05E+00
**Average**	1.12E-01	2.50E-01	4.64E-01	6.66E-01	4.93E+00
**STD**	1.12E-02	2.49E-02	4.63E-02	6.65E-02	4.92E-01

Table S8. Retained REE mass per 1 g of natural sand for batch and column experiments with brackish water.

**Natural sand Brackish water**	Retained REE (µg REE / g soil)				
	Column exp				Batch exp
	**20 PV**	**50 PV**	**100 PV**	**150 PV**	**8 Days**
La	9.85E-02	2.37E-01	4.47E-01	6.60E-01	4.32E+00
Ce	1.51E-01	3.63E-01	6.86E-01	1.01E+00	6.63E+00
Pr	1.16E-01	2.79E-01	5.27E-01	7.79E-01	5.09E+00
Nd	1.19E-01	2.86E-01	5.40E-01	7.99E-01	5.22E+00
Sm	1.24E-01	2.97E-01	5.61E-01	8.30E-01	5.42E+00
Eu	1.23E-01	2.95E-01	5.56E-01	8.23E-01	5.38E+00
Gd	1.19E-01	2.87E-01	5.42E-01	8.01E-01	5.24E+00
Tb	1.18E-01	2.84E-01	5.36E-01	7.93E-01	5.18E+00
Dy	1.06E-01	2.55E-01	4.82E-01	7.13E-01	4.66E+00
Ho	1.16E-01	2.79E-01	5.27E-01	7.79E-01	5.09E+00
Er	1.12E-01	2.70E-01	5.10E-01	7.54E-01	4.93E+00
Tm	1.13E-01	2.72E-01	5.14E-01	7.59E-01	4.96E+00
Yb	1.10E-01	2.65E-01	5.00E-01	7.39E-01	4.83E+00
Lu	1.09E-01	2.63E-01	4.96E-01	7.33E-01	4.79E+00
**Average**	1.17E-01	2.81E-01	5.30E-01	7.84E-01	5.12E+00
**STD**	1.20E-02	2.87E-02	5.42E-02	8.02E-02	5.24E-01

Table S9. Retained REE mass per 1 g of low-carbonate calcareous sandstone 1 for batch and column experiments with fresh water.

**Low-carbonate calcareous sandstone 1 Fresh water**	Retained REE (µg REE / g soil)				
	Column exp				Batch exp
	**20 PV**	**50 PV**	**100 PV**	**150 PV**	**8 Days**
La	8.28E-02	1.94E-01	3.73E-01	5.40E-01	3.44E+00
Ce	1.73E-01	4.04E-01	7.78E-01	1.13E+00	7.18E+00
Pr	1.12E-01	2.62E-01	5.05E-01	7.32E-01	4.66E+00
Nd	1.22E-01	2.84E-01	5.48E-01	7.94E-01	5.05E+00
Sm	1.28E-01	3.00E-01	5.77E-01	8.36E-01	5.32E+00
Eu	1.28E-01	3.00E-01	5.79E-01	8.39E-01	5.34E+00
Gd	1.20E-01	2.80E-01	5.40E-01	7.82E-01	4.98E+00
Tb	1.33E-01	3.11E-01	6.00E-01	8.69E-01	5.53E+00
Dy	1.36E-01	3.19E-01	6.14E-01	8.90E-01	5.66E+00
Ho	1.35E-01	3.16E-01	6.09E-01	8.83E-01	5.62E+00
Er	1.40E-01	3.27E-01	6.30E-01	9.12E-01	5.81E+00
Tm	1.48E-01	3.46E-01	6.66E-01	9.65E-01	6.14E+00
Yb	1.51E-01	3.54E-01	6.81E-01	9.87E-01	6.28E+00
Lu	1.55E-01	3.62E-01	6.97E-01	1.01E+00	6.43E+00
**Average**	1.33E-01	3.11E-01	6.00E-01	8.69E-01	5.53E+00
**STD**	2.14E-02	5.01E-02	9.66E-02	1.40E-01	8.91E-01

Table S10. Retained REE mass per 1 g of low-carbonate calcareous sandstone 1 for batch and column experiments with brackish water.

**Low-carbonate calcareous sandstone 1 Brackish water**	Retained REE (µg REE / g soil)				
	Column exp				Batch exp
	**20 PV**	**50 PV**	**100 PV**	**150 PV**	**8 Days**
La	7.16E-02	1.78E-01	3.45E-01	4.92E-01	3.30E+00
Ce	1.57E-01	3.89E-01	7.55E-01	1.08E+00	7.22E+00
Pr	1.02E-01	2.54E-01	4.93E-01	7.03E-01	4.71E+00
Nd	1.22E-01	3.03E-01	5.87E-01	8.37E-01	5.61E+00
Sm	1.19E-01	2.96E-01	5.74E-01	8.19E-01	5.49E+00
Eu	1.21E-01	3.00E-01	5.81E-01	8.29E-01	5.56E+00
Gd	1.23E-01	3.04E-01	5.90E-01	8.42E-01	5.64E+00
Tb	1.35E-01	3.36E-01	6.51E-01	9.29E-01	6.23E+00
Dy	1.36E-01	3.37E-01	6.54E-01	9.33E-01	6.25E+00
Ho	1.36E-01	3.38E-01	6.56E-01	9.36E-01	6.27E+00
Er	1.44E-01	3.57E-01	6.92E-01	9.87E-01	6.62E+00
Tm	1.48E-01	3.68E-01	7.14E-01	1.02E+00	6.83E+00
Yb	1.53E-01	3.79E-01	7.36E-01	1.05E+00	7.04E+00
Lu	1.44E-01	3.58E-01	6.94E-01	9.90E-01	6.64E+00
**Average**	1.29E-01	3.21E-01	6.23E-01	8.89E-01	5.96E+00
**STD**	2.25E-02	5.58E-02	1.08E-01	1.54E-01	1.03E+00

Table S11. Retained REE mass per 1 g of low-carbonate calcareous sandstone 2 for batch and column experiments with fresh water.

Low-carbonate calcareous sandstone 2 Fresh water	Retained REE (µg REE / g soil)				
	Column exp				Batch exp
	**20 PV**	**50 PV**	**100 PV**	**150 PV**	**8 Days**
La	8.28E-02	1.91E-01	3.68E-01	5.35E-01	3.32E+00
Ce	1.54E-01	3.54E-01	6.83E-01	9.94E-01	6.17E+00
Pr	1.10E-01	2.53E-01	4.89E-01	7.12E-01	4.42E+00
Nd	1.21E-01	2.79E-01	5.38E-01	7.83E-01	4.86E+00
Sm	1.26E-01	2.90E-01	5.61E-01	8.16E-01	5.06E+00
Eu	1.26E-01	2.91E-01	5.62E-01	8.17E-01	5.07E+00
Gd	1.20E-01	2.76E-01	5.33E-01	7.76E-01	4.82E+00
Tb	1.28E-01	2.96E-01	5.71E-01	8.30E-01	5.15E+00
Dy	1.32E-01	3.03E-01	5.86E-01	8.52E-01	5.29E+00
Ho	1.31E-01	3.01E-01	5.82E-01	8.46E-01	5.25E+00
Er	1.35E-01	3.10E-01	5.99E-01	8.71E-01	5.40E+00
Tm	1.40E-01	3.23E-01	6.23E-01	9.06E-01	5.62E+00
Yb	1.43E-01	3.30E-01	6.37E-01	9.26E-01	5.75E+00
Lu	1.45E-01	3.35E-01	6.46E-01	9.40E-01	5.83E+00
**Average**	1.28E-01	2.95E-01	5.70E-01	8.29E-01	5.14E+00
**STD**	1.73E-02	3.98E-02	7.69E-02	1.12E-01	6.94E-01

Table S12. Retained REE mass per 1 g of low-carbonate calcareous sandstone 2 for batch and column experiments with brackish water.

**Low-carbonate calcareous sandstone 2 Brackish water**	Retained REE (µg REE / g soil)				
	Column exp				Batch exp
	**20 PV**	**50 PV**	**100 PV**	**150 PV**	**8 Days**
La	7.75E-02	1.94E-01	3.82E-01	5.68E-01	3.38E+00
Ce	1.43E-01	3.58E-01	7.06E-01	1.05E+00	6.25E+00
Pr	1.02E-01	2.54E-01	5.01E-01	7.43E-01	4.43E+00
Nd	1.16E-01	2.90E-01	5.74E-01	8.51E-01	5.08E+00
Sm	1.18E-01	2.96E-01	5.84E-01	8.67E-01	5.17E+00
Eu	1.24E-01	3.08E-01	6.09E-01	9.05E-01	5.39E+00
Gd	1.21E-01	3.03E-01	5.98E-01	8.88E-01	5.29E+00
Tb	1.33E-01	3.33E-01	6.57E-01	9.76E-01	5.82E+00
Dy	1.36E-01	3.40E-01	6.71E-01	9.96E-01	5.94E+00
Ho	1.38E-01	3.45E-01	6.81E-01	1.01E+00	6.03E+00
Er	1.41E-01	3.53E-01	6.97E-01	1.03E+00	6.17E+00
Tm	1.54E-01	3.84E-01	7.59E-01	1.13E+00	6.72E+00
Yb	1.59E-01	3.97E-01	7.85E-01	1.17E+00	6.95E+00
Lu	1.60E-01	4.00E-01	7.89E-01	1.17E+00	6.99E+00
**Average**	1.30E-01	3.25E-01	6.43E-01	9.54E-01	5.69E+00
**STD**	2.28E-02	5.69E-02	1.12E-01	1.67E-01	9.94E-01

Table S13. Retained REE mass per 1 g of high-carbonate calcareous sandstone for batch and column experiments with fresh water.

**High-carbonate calcareous sandstone Fresh water**	Retained REE (µg REE / g soil)				
	Column exp				Batch exp
	**20 PV**	**50 PV**	**100 PV**	**150 PV**	**8 Days**
La	8.28E-02	2.07E-01	3.66E-01	5.97E-01	4.48E+00
Ce	2.20E-01	5.49E-01	9.70E-01	1.58E+00	1.19E+01
Pr	1.09E-01	2.73E-01	4.82E-01	7.87E-01	5.91E+00
Nd	1.20E-01	3.00E-01	5.30E-01	8.66E-01	6.51E+00
Sm	1.27E-01	3.16E-01	5.59E-01	9.12E-01	6.85E+00
Eu	1.26E-01	3.15E-01	5.56E-01	9.08E-01	6.82E+00
Gd	1.21E-01	3.02E-01	5.34E-01	8.72E-01	6.55E+00
Tb	1.37E-01	3.42E-01	6.03E-01	9.85E-01	7.40E+00
Dy	1.44E-01	3.61E-01	6.37E-01	1.04E+00	7.81E+00
Ho	1.44E-01	3.60E-01	6.36E-01	1.04E+00	7.80E+00
Er	1.51E-01	3.77E-01	6.66E-01	1.09E+00	8.17E+00
Tm	1.60E-01	4.01E-01	7.08E-01	1.16E+00	8.69E+00
Yb	1.67E-01	4.18E-01	7.37E-01	1.20E+00	9.04E+00
Lu	1.78E-01	4.45E-01	7.85E-01	1.28E+00	9.63E+00
**Average**	1.42E-01	3.55E-01	6.26E-01	1.02E+00	7.68E+00
**STD**	3.33E-02	8.32E-02	1.47E-01	2.40E-01	1.80E+00

Table S14. Retained REE mass per 1 g of high-carbonate calcareous sandstone for batch and column experiments with brackish water.

**High-carbonate calcareous sandstone Brackish water**	Retained REE (µg REE / g soil)				
	Column exp				Batch exp
	**20 PV**	**50 PV**	**100 PV**	**150 PV**	**8 Days**
La	8.05E-02	2.01E-01	4.00E-01	5.81E-01	4.46E+00
Ce	1.45E-01	3.63E-01	7.23E-01	1.05E+00	8.05E+00
Pr	1.05E-01	2.62E-01	5.21E-01	7.56E-01	5.80E+00
Nd	1.14E-01	2.84E-01	5.65E-01	8.21E-01	6.30E+00
Sm	1.15E-01	2.89E-01	5.74E-01	8.34E-01	6.40E+00
Eu	1.24E-01	3.10E-01	6.16E-01	8.95E-01	6.86E+00
Gd	1.20E-01	2.99E-01	5.94E-01	8.63E-01	6.62E+00
Tb	1.37E-01	3.43E-01	6.82E-01	9.91E-01	7.60E+00
Dy	1.64E-01	4.11E-01	8.17E-01	1.19E+00	9.10E+00
Ho	1.48E-01	3.70E-01	7.35E-01	1.07E+00	8.20E+00
Er	1.51E-01	3.77E-01	7.51E-01	1.09E+00	8.36E+00
Tm	1.60E-01	4.01E-01	7.97E-01	1.16E+00	8.88E+00
Yb	1.62E-01	4.04E-01	8.04E-01	1.17E+00	8.96E+00
Lu	1.66E-01	4.14E-01	8.24E-01	1.20E+00	9.18E+00
**Average**	1.35E-01	3.38E-01	6.72E-01	9.76E-01	7.48E+00
**STD**	2.60E-02	6.49E-02	1.29E-01	1.88E-01	1.44E+00

Table S15. Retained REE mass per 1 g of red loamy sand for batch and column experiments with fresh water.

**Red loamy sand Fresh water**	Retained REE (µg REE / g soil)				
	Column exp				Batch exp
	**20 PV**	**50 PV**	**100 PV**	**150 PV**	**8 Days**
La	7.66E-02	1.87E-01	3.56E-01	5.33E-01	4.41E+00
Ce	1.68E-01	4.10E-01	7.79E-01	1.17E+00	9.67E+00
Pr	1.04E-01	2.54E-01	4.82E-01	7.24E-01	5.99E+00
Nd	1.19E-01	2.91E-01	5.52E-01	8.29E-01	6.85E+00
Sm	1.24E-01	3.02E-01	5.74E-01	8.62E-01	7.13E+00
Eu	1.19E-01	2.90E-01	5.51E-01	8.26E-01	6.84E+00
Gd	1.20E-01	2.92E-01	5.55E-01	8.32E-01	6.88E+00
Tb	1.29E-01	3.15E-01	5.99E-01	8.98E-01	7.43E+00
Dy	1.39E-01	3.39E-01	6.45E-01	9.67E-01	8.00E+00
Ho	1.37E-01	3.34E-01	6.35E-01	9.53E-01	7.89E+00
Er	1.40E-01	3.41E-01	6.48E-01	9.73E-01	8.05E+00
Tm	1.43E-01	3.48E-01	6.62E-01	9.93E-01	8.21E+00
Yb	1.57E-01	3.82E-01	7.27E-01	1.09E+00	9.02E+00
Lu	1.55E-01	3.78E-01	7.18E-01	1.08E+00	8.91E+00
**Average**	1.31E-01	3.19E-01	6.06E-01	9.09E-01	7.52E+00
**STD**	2.33E-02	5.69E-02	1.08E-01	1.62E-01	1.34E+00

Table S16. Retained REE mass per 1 g of red loamy sand for batch and column experiments with brackish water.

**Red Loamy sand Brackish water**	Retained REE (μg REE / g soil)				
	Column exp				Batch exp
	**20 PV**	**50 PV**	**100 PV**	**150 PV**	**8 Days**
La	1.03E-01	2.57E-01	5.03E-01	7.61E-01	6.01E+00
Ce	1.13E-01	2.84E-01	5.55E-01	8.39E-01	6.62E+00
Pr	1.01E-01	2.52E-01	4.94E-01	7.47E-01	5.90E+00
Nd	1.11E-01	2.78E-01	5.44E-01	8.22E-01	6.50E+00
Sm	1.09E-01	2.71E-01	5.32E-01	8.03E-01	6.34E+00
Eu	1.09E-01	2.73E-01	5.35E-01	8.08E-01	6.38E+00
Gd	1.20E-01	2.99E-01	5.85E-01	8.84E-01	6.98E+00
Tb	1.12E-01	2.79E-01	5.46E-01	8.25E-01	6.52E+00
Dy	1.15E-01	2.89E-01	5.65E-01	8.54E-01	6.75E+00
Ho	1.19E-01	2.98E-01	5.84E-01	8.82E-01	6.97E+00
Er	1.19E-01	2.99E-01	5.85E-01	8.83E-01	6.98E+00
Tm	1.21E-01	3.02E-01	5.92E-01	8.94E-01	7.06E+00
Yb	1.19E-01	2.97E-01	5.81E-01	8.77E-01	6.93E+00
Lu	1.24E-01	3.09E-01	6.05E-01	9.13E-01	7.22E+00
**Average**	1.14E-01	2.85E-01	5.58E-01	8.42E-01	6.65E+00
**STD**	6.85E-03	1.71E-02	3.35E-02	5.06E-02	4.00E-01

Figures

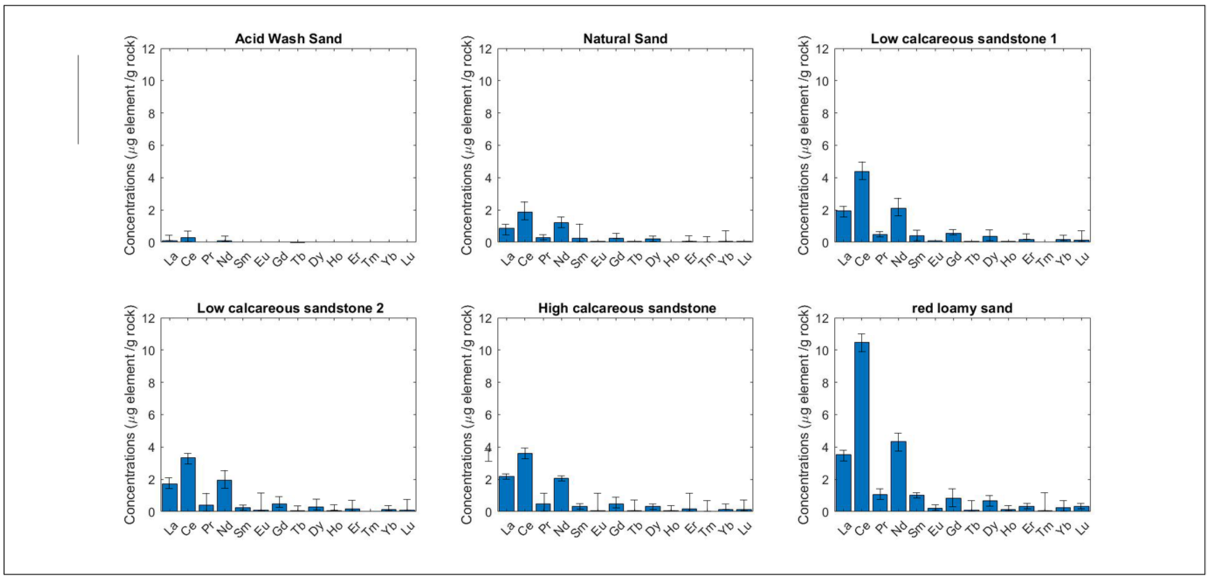

Figure S1. Concentrations of REEs in the different coastal aquifer materials.

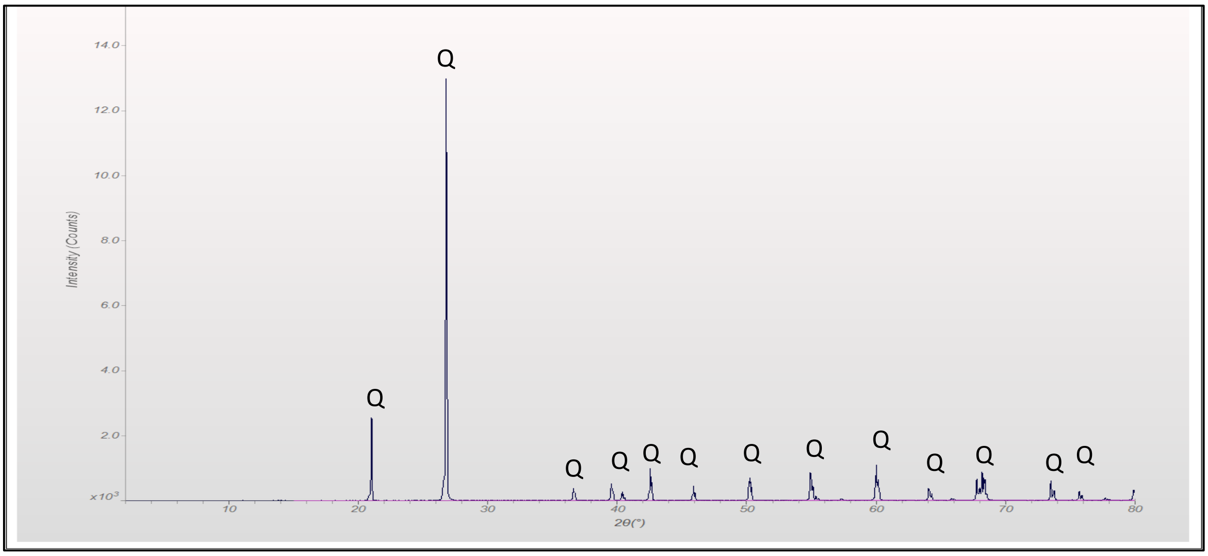

Figure S2. XRD of acid-wash sand bulk sample. Q=quartz

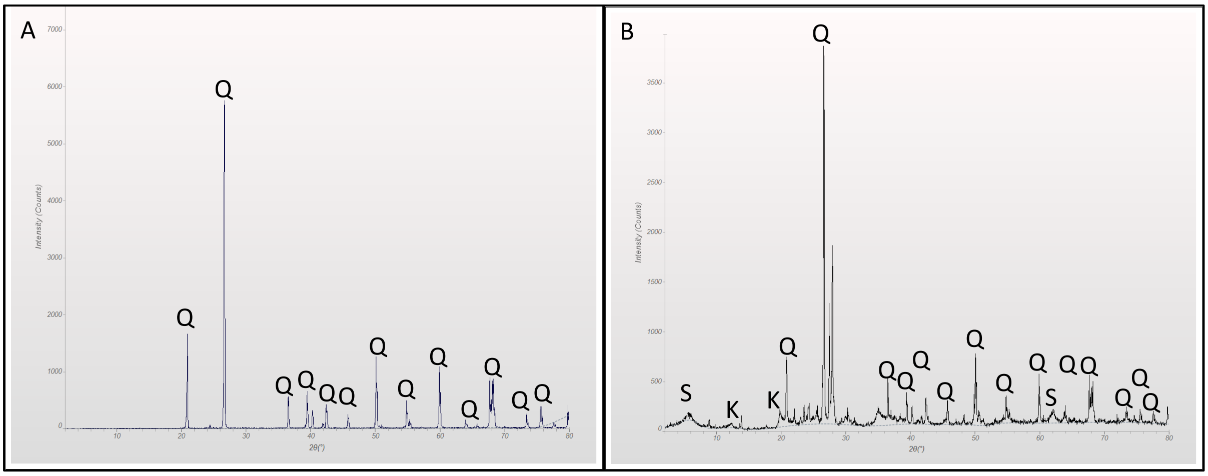

Figure S3. XRD of natural sand sample. A) Bulk. B) Small grain size fraction. Q = quartz. S = smectite. K = kaolin.

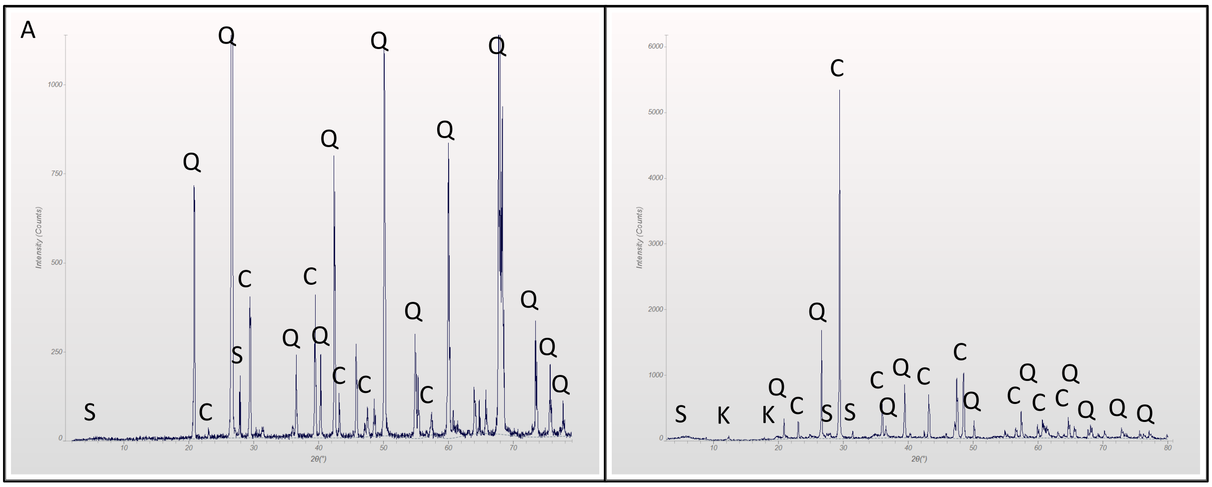

Figure S4. XRD of low-carbonate calcareous sandstone 1. A) Bulk. B) Small grain size fraction. Q = quartz. S = smectite. K = kaolin. C = calcite.

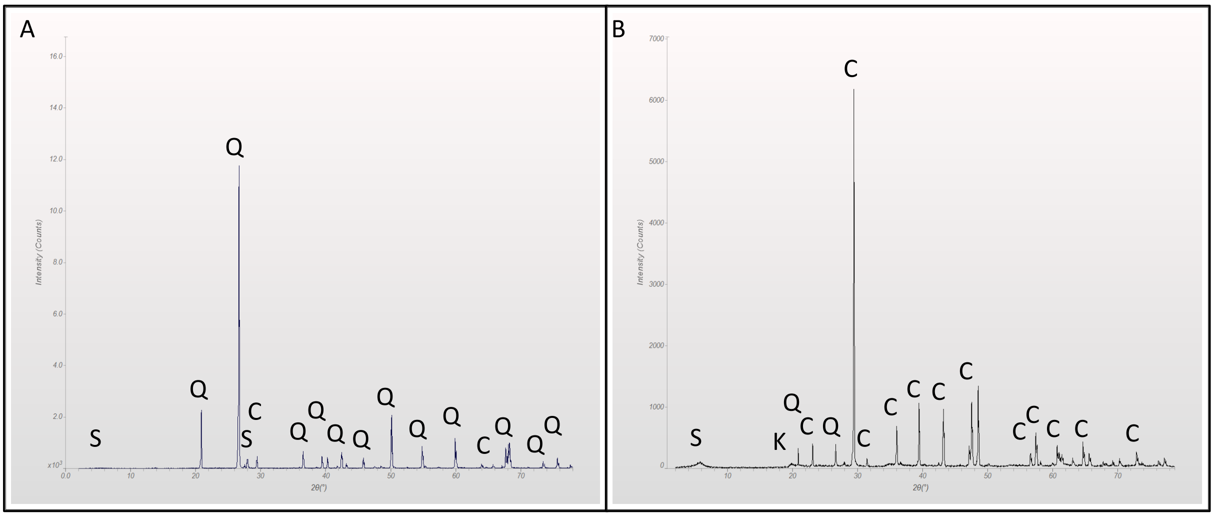

Figure S5. XRD of low-carbonate calcareous sandstone 2. A) Bulk. B) small grain size fraction. Q= quartz. S= smectite. K=kaolin. C=calcite.

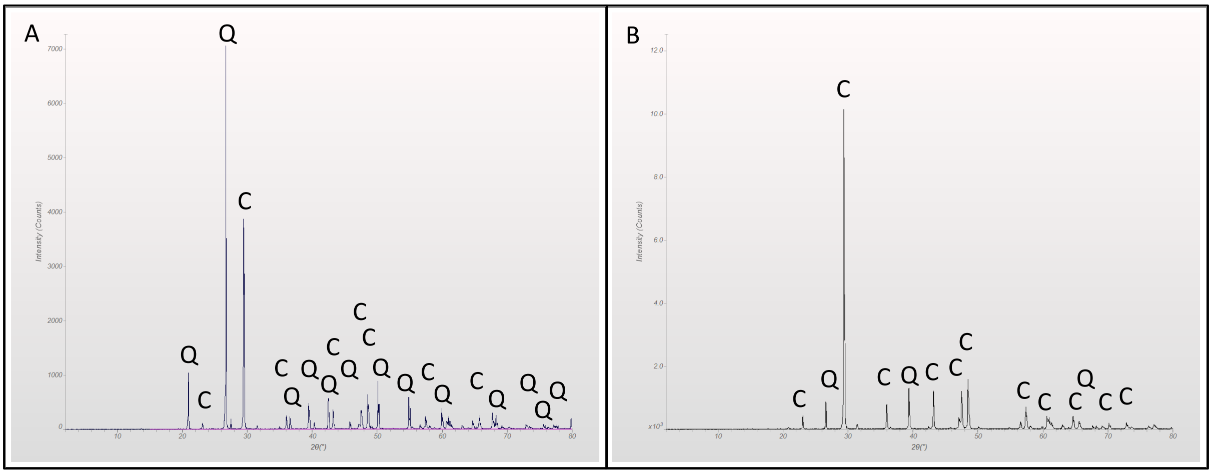

Figure S6. XRD of high-carbonate calcareous sandstone. A) Bulk. B) Small grain size fraction. Q = quartz. C = calcite.

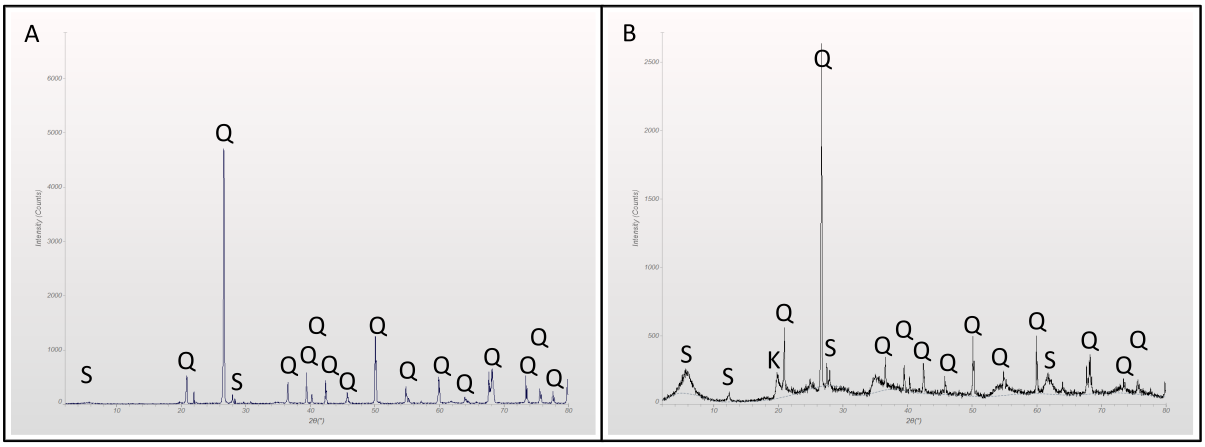

Figure S7. XRD of red loamy sand. A) Bulk. B) Small grain size fraction. Q = quartz. S = smectite. K = kaolin.

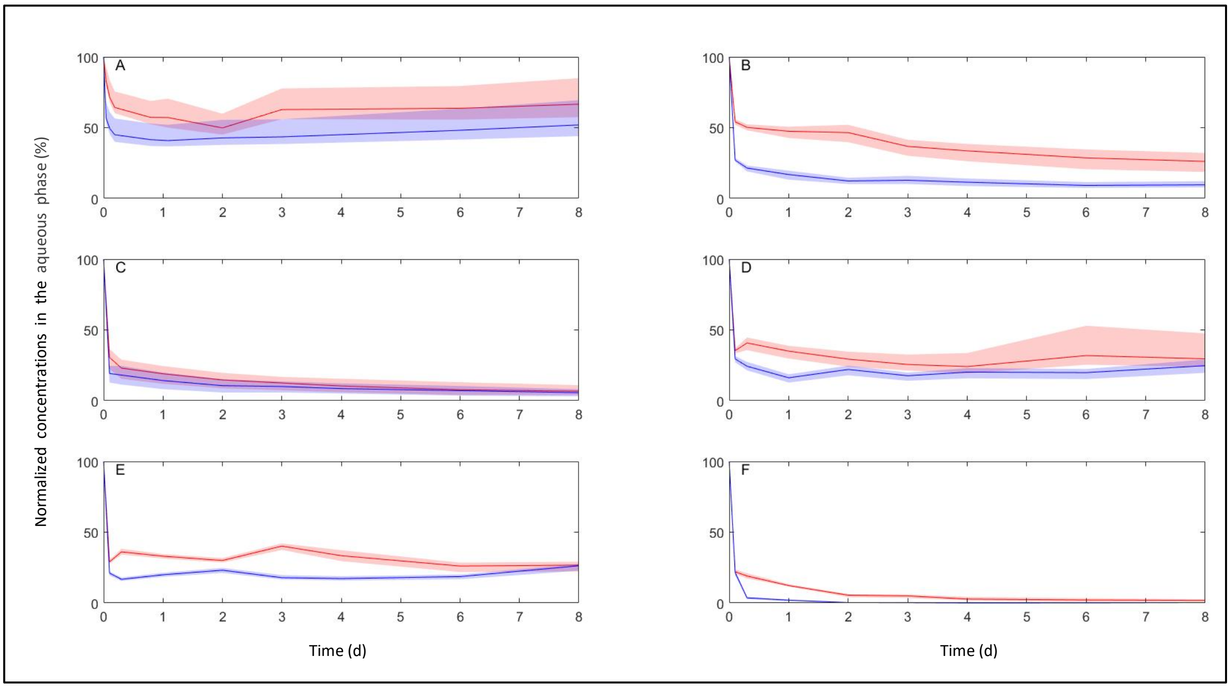

Figure S8. Adsorption curves of REE in different Coastal Aquifer materials and salinities. A. acid-wash sand, B. natural sand. C. low-carbonate calcareous sandstone 1. D. low-carbonate calcareous sandstone 2. E. high-carbonate calcareous sandstone F. red sandy soil. Red line: REE average in fresh water conditions. Red background: REE distribution in fresh water conditions. Blue line: REE average in brackish water conditions. Blue background: REE distribution in brackish water conditions.

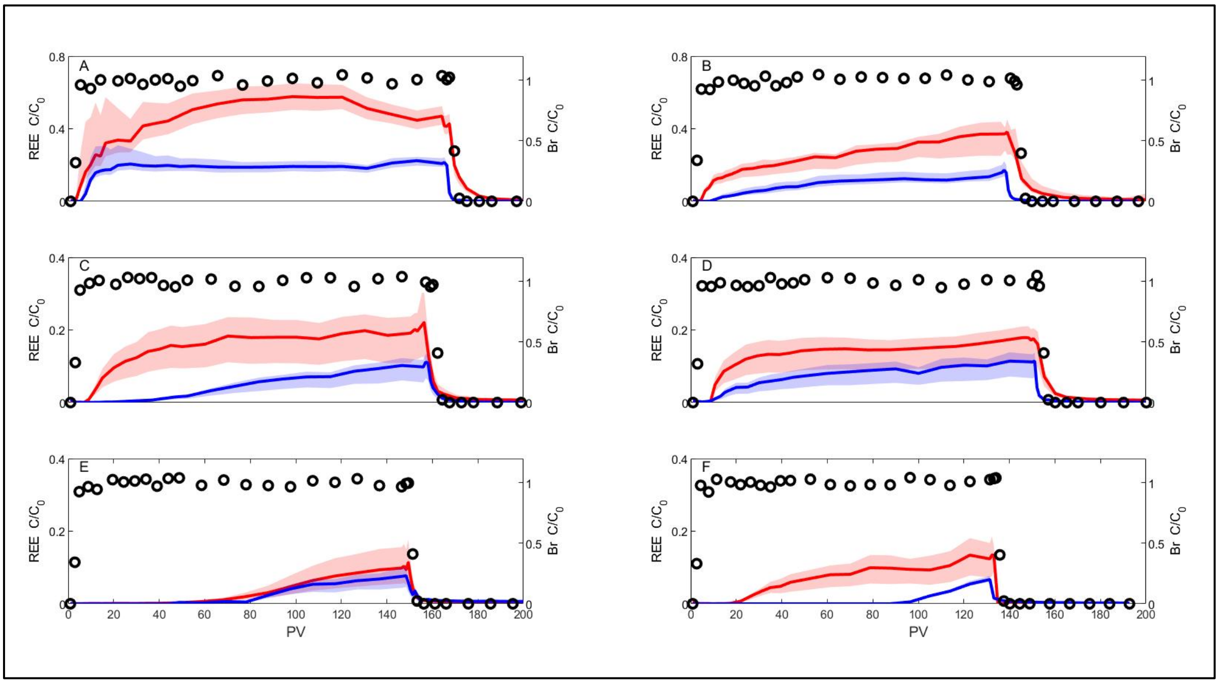

Figure S9. Breakthrough curve measurements of REEs (average concentration) and Br tracer in different Coastal Aquifer materials and salinities. Note: different y-axis values. A. acid-wash sand, B. natural sand. C. low-carbonate calcareous sandstone 1. D. low-carbonate calcareous sandstone 2. E. high-carbonate calcareous sandstone F. red sandy soil. Red line: REE average in fresh water conditions (IS=2.5x10-3). Red background: REE distribution in fresh water conditions. Blue line: REE average in brackish water conditions (IS=2.5x10-2). Blue background: REE distribution in brackish water conditions. Black circles: Br tracer.

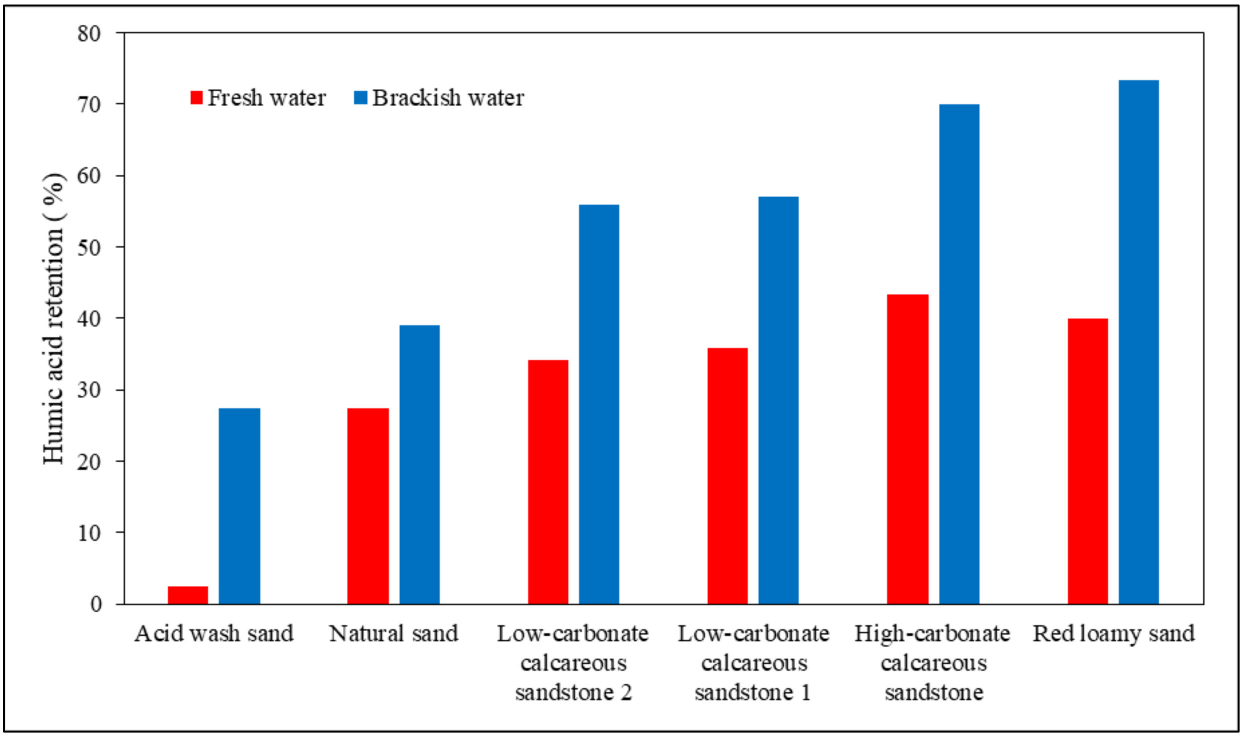

Figure S10. Humic acid retention on the different coastal aquifer materials in fresh and brackish water conditions. Red bars: fresh water conditions (Ionic strength=2.5×10-3M). Blue bars: brackish water conditions (Ionic strength=2.5×10-2M).

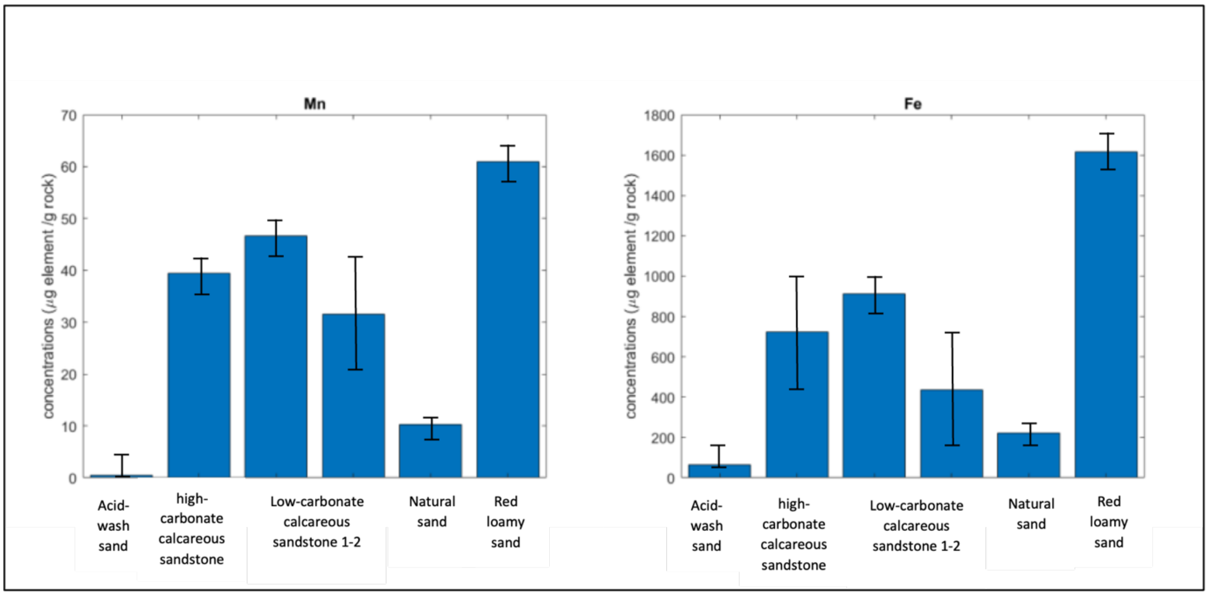

Figure S11. Concentrations of Mn and Fe in the different coastal aquifer materials.
